# Synthesis, surface activities, aggregation properties and oil washing performances of novel cardanol-based surfactants

**DOI:** 10.1371/journal.pone.0344255

**Published:** 2026-04-22

**Authors:** Weiyang Liu, Congying Lu, Xin Zhao, Zuxi Zhang, Ruihong Huang, Lingxiao Fan, Wei Ding

**Affiliations:** 1 Key Laboratory of Enhanced Oil Recovery, Ministry of Education, College of Petroleum Engineering, Northeast Petroleum University, Daqing, Heilongjiang, China; 2 Heilongjiang Key Laboratory of Oilfield Applied Chemistry and Technology of Daqing Normal University, Daqing, Heilongjiang, China; 3 Exploration and Development Research Institute of Daqing Oilfield Recovery Research Room 2, Daqing, Heilongjiang, China; 4 Northeast Petroleum University, College of Chemistry and Chemical Engineering, Oil and Gas Chemical Technology Provincial Key Laboratory, Daqing, Heilongjiang, China; Tribhuvan University, NEPAL

## Abstract

A mixed cardanol sulfonate surfactant (MYBS) of disubstituted styrene unsaturated cardanol sulfonate (2,4-YBS) and monosubstituted styrene unsaturated cardanol sulfonate (2-YBS) was synthesized by optimizing the optimal synthesis conditions of the three-step reaction of alkylation, sulfonation and neutralization. The molar percentages of the two are about 70%: 30%. Its structure was characterized by gas chromatography-mass spectrometry and infrared spectroscopy. At 25 °C, the surface tension of MYBS at critical micelle concentration (γCMC) and critical micelle concentration (CMC) was 33.32 mN/m and 0.676 mmol/L, respectively, which was better than that of traditional anionic surfactant (SDBS) and cardanol sulfonate surfactant (CDS). The thermodynamic parameters obtained from the conductivity measurements confirmed that the micellization process was a spontaneous and entropy-driven process. Dynamic light scattering test and transmission electron microscopy showed that MYBS molecules could form small spherical micelles of 10 nm-30 nm, which was consistent with the calculation results of critical packing theory. At the same time, the oil washing experiment confirmed that the oil washing efficiency can reach 73%, which provides technical support for the application of this kind of surfactant in improving oil recovery.

## 1. Introduction

Cashew nut shell liquid surfactant (CNSL) is a kind of environmentally friendly material based on natural plant resources [1,2]. Due to its unique molecular structure and excellent properties, CNSL has shown broad application prospects in many fields such as detergents, emulsifiers, and drug delivery systems [3–5]. With the improvement of the concept of sustainable development and the awareness of environmental protection, green chemistry has received extensive attention in the research of materials and chemical products [6–8]. The development of green surfactants from natural sources has become an important research direction in the field of chemistry. The cardanol surfactant coincides with this green development trend. With its natural properties and excellent application performance, it occupies a core position in the research and practical application of green surfactants.

Cardanol is obtained from the extract of cashew nut shell by heating decarboxylation, which has good use in many fields. Cardanol has the unique advantages of renewable raw materials, low price, strong biodegradability, low toxicity, and a wide application range [9]; at the same time, the structure of cardanol contains not only unsaturated long alkane chains with 0–3 C = C double bonds, but also phenolic hydroxyl groups. Its benzene ring also contains four reaction sites, which are highly chemically modifiable. In recent years, a variety of cardanol-based chemical products with excellent properties have been successfully synthesized by extensive research, such as phenolic resin, epoxy resin, coatings, dyes, plasticizers, and surfactants. These products have extremely important development and utilization value [10–16]. Recent studies have highlighted the importance of interfacial structure and microenvironmental effects in aggregation and transport phenomena across fluid, colloidal, and interdisciplinary systems [17–21]. However, most previous research has focused on modification of the hydroxyl group, and surfactants functionalized on the benzene ring remain less explored, representing a significant challenge.

In the past ten years, with the deepening of the research on cardanol surfactant, researchers have gained a deeper understanding of it. The related research mainly focuses on the modification of cardanol hydroxyl groups. Anionic [22], cationic [23], non-ionic [24], zwitterionic [25], and Gemini surfactants [26] have been successfully synthesized. However, there are few reports on the synthesis of surfactants modified on the benzene ring of cardanol, which is mainly due to the great challenges in synthesis and structural analysis with four active hydrogen sites on the benzene ring. Khatib et al. [27] studied the anionic surfactant with two carboxylic acid groups by using cardanolic acid as a raw material, retaining the carboxylic acid on the cardanol molecule and introducing carboxylic acid on the phenolic hydroxyl group. The CMC value of the product is 0.177 mmol/L, which has good surface activity. Gooßen et al. [28] studied the use of cardanol as a raw material, 3-pentadecylphenol after catalytic hydrogenation with palladium carbon, that is, 3-pentadecylcyclohexane-1-ol, and chlorotoluene through quaternary ammonium salt reaction to obtain cardanol cationic surfactant; the CMC of the molecule is minimal. Han et al. [29] used epichlorohydrin as a connecting group to modify the hydroxyl end of cardanol to ethylene oxide, and then reacted with tertiary amines to obtain a series of products. The obtained series of products has lower CMC values, surface tension is about 20mN/m, and surface activity is higher. Faye et al. [6] synthesized a cardanol amphoteric surfactant with a sulfonic acid group and a cationic ammonium group for the first time. This surfactant has a higher CMC value and lower surface tension than pure anionic and cationic surfactants. Li et al. [30] synthesized a cardanol Gemini anionic surfactant with a hydrophilic chain of polyoxyethylene ether acetic acid. The surfactant has a lower surface tension and CMC value, and as the number of EO increases, micellization is easier to form.

In order to further deepen the research on the modification of the benzene ring of cardanol and the synthesis of surfactants, this paper is based on the previous research on the alkylation reaction of styrene, α-methylstyrene and phenol by P. Sh. Mamedova [31], Hyeonjun Yun [32], Zhang Jinlong [33], Jiang Yongbo [34], Zou Donglei [35] and other researchers. Based on the previous research on the alkylation reaction of styrene, α-methylstyrene, and phenol, a new type of structural cardanol sulfonate surfactant was synthesized by alkylation reaction, sulfonation, and neutralization reaction of green biomass cardanol and styrene. The new structure of cardanol sulfonate surfactant is a type of green and sustainable surfactant, whichhas significant development potential and application prospects in enhancing oil recovery. Based on the hypothesis that the hydrophobic structure of the multi-benzene ring can improve the surface activity and enhance the thermodynamic stability of the micelles. In this study, by designing the synthesis conditions and optimizing the optimal synthesis parameters, a mixed cardanol sulfonate surfactant with 2,4-YBS product as the main body and 2-YBS was synthesized by introducing a styrene group as a hydrophobic group and a sulfonic acid group as a hydrophilic group on the benzene ring of cardanol. The surface activity, thermodynamic parameters, aggregation behavior, and other physical and chemical properties of MYBS surfactants were investigated by means of surface tension, conductivity, dynamic light scattering, transmission electron microscopy, and critical stacking parameter calculation. The interfacial properties and self-assembly behavior were characterized. In addition, the oil washing effect was investigated by an oil washing experiment, and the results showed that the oil washing efficiency was 73%.

## 2. Materials and methods

### 2.1. Reagents and instruments

Cardanol (302.2 g/mol, industrial grade, Jinan Renyuan Chemical Co., Ltd.); styrene (> 98%), analytically pure, Tianjin Kemiou Chemical Reagent Co., Ltd.; chlorosulfonic acid, industrial grade, Shanghai Sinopharm Reagent Co., Ltd.; methanesulfonic acid (> 98%), dichloromethane (> 99%), sodium hydroxide (> 98%), petroleum ether (> 98%), anhydrous ethanol (> 99.5%), were analytically pure, Tianjin Damao Chemical Reagent Co., Ltd.; all solutions are prepared using ultrapure water (18.2 MΩcm); quartz sand (80–100 mesh), analytically pure, Tianjin Damao Chemical Reagent Co., Ltd., oil samples from Liaohe Oilfield, China.

C63 organic synthesis instrument, Tianjin Glass Instrument Factory; dF-101B type digital display heat collecting magnetic stirrer, Zhengzhou Dongsheng Instrument and Equipment Co., Ltd.; R201B rotary evaporator, Shanghai Shensheng Biotechnology Co., Ltd.; DZF vacuum drying oven, Guangzhou Kangheng Instrument Co., Ltd.; 0.2 mL drop volume capillary, Huang Jianbin research group of Peking University; 100mL washing oil bottle, Tianjin Glass Instrument Factory; 7890B-5977A gas chromatography-mass spectrometer (Agilent, USA); Tensor27 Fourier Transform Infrared Spectrometer, Bruker, USA; DDSJ-308F Conductivity Meter, Shanghai LeiMagnetic Instrument Co., Ltd.; F-4600 fluorescence spectrophotometer (Hitachi High-Technologies Corp., Tokyo, Japan); Zetasizer Nano ZS90 Dynamic Light Scattering Instrument, Malvern, UK; JEM-2100PLUS transmission electron microscope (JEOL, Japan).

### 2.2. The synthesis of MYBS

**Fig 1:**
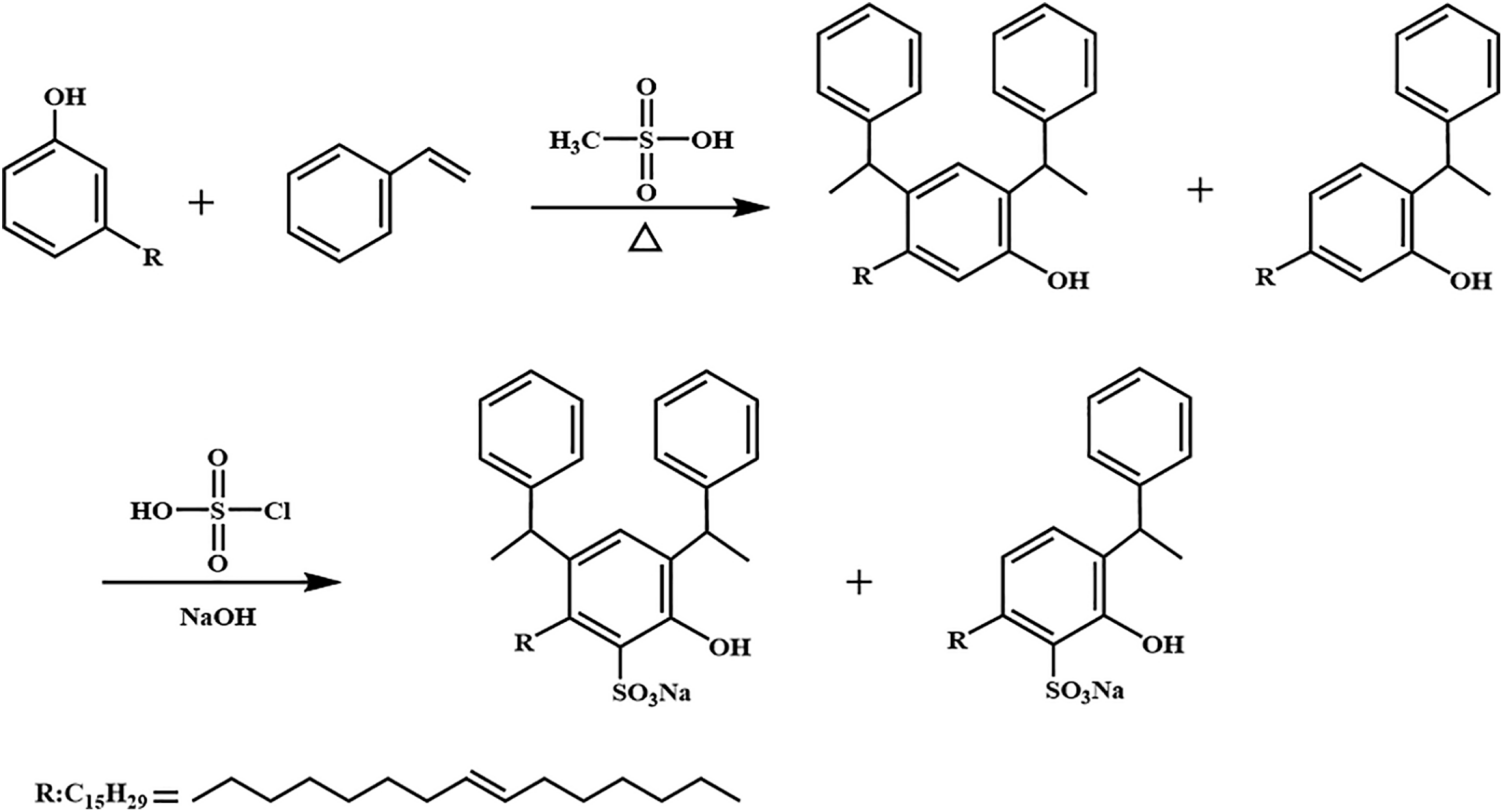
Synthesis route of mixed cashew phenol sulfonate surfactant MYBS with 2,4-YBS and 2-YBS.

**Fig 2:**
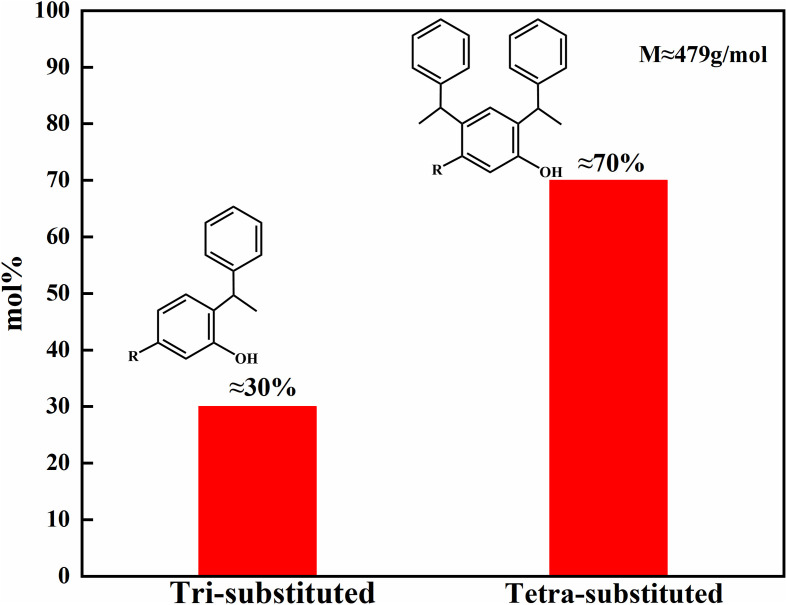
Gas chromatography-mass spectrometry (GC-MS) spectrum of styrene unsaturated cardanol alkylation product.

**Fig 3:**
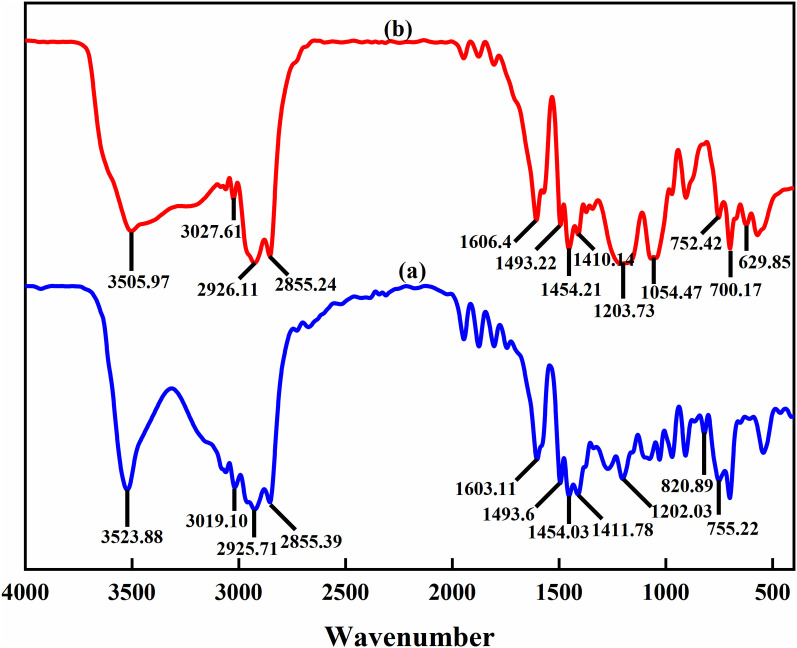
FTIR spectra of styryl unsaturated cardanol alkylation products and styryl unsaturated cardanol sulfonates.

The mixed cardanol sulfonate surfactants of bis-substituted styrene unsaturated cardanol sulfonate (2,4-YBS) and mono-substituted styrene unsaturated cardanol sulfonate (2-YBS) were synthesized by a simple three-step method. Firstly, the molar ratio of cardanol to styrene was 1: 2.1, and the amount of methanesulfonic acid catalyst was 1.65% of the total mass. Cardanol (45.3 g, 0.15 mol) and methanesulfonic acid catalyst (1.288 g) were added to a dry 250 mL four-necked flask, stirred, and heated to 70 °C. Then, excess styrene (32.76 g, 0.315 mol) was added to the solution with a constant pressure drop funnel drop, and the reaction mixture was then stirred at 70 °C for 2 h. Finally, the reaction mixture was transferred to the separation funnel, and it was washed with distilled water multiple times until the pH value of the reaction mixture was neutral. The upper solution was taken to remove distilled water and residual styrene by vacuum distillation to obtain an intermediate (dark brown liquid). The intermediate product was sulfonated with chlorosulfonic acid. The intermediate product (47.9 g, 0.1 mol) and dichloromethane (150 mL) were added to the three-mouth flask, and the chlorosulfonic acid (12.82 g, 0.11 mol) was dropped. The reaction mixture was kept below 10 °C in the ice bath. After the drop, the temperature was raised to 25 °C, and the stirring reaction was continued for 2 h. The dichloromethane solvent was removed by vacuum distillation to obtain the sulfonated product. Then the sulfonated product was dissolved in anhydrous ethanol (50 mL), and a 20% NaOH solution for neutralization reaction, and the pH value of the product was adjusted to 8–10. The anhydrous ethanol and water were removed by vacuum distillation to obtain the brown solid product. MYBS is treated as a mixed surfactant system obtained from synthesis rather than a conventional binary mixture of two independent surfactants. The synthetic route is shown in Fig 1, and the intermediate product gas-mass spectrometry chromatography is shown in Fig 2, and the final product infrared spectrum is shown in Fig 3.

From Fig 2, it can be seen that the alkylation product is a mixture. By optimizing the optimal reaction conditions, the core goal of synthesizing bis-substituted styrene unsaturated cardanol alkylation product as the main product is met. Finally, the molar percentage of bis-substituted styrene unsaturated cardanol alkylation product is about 70%, the molar percentage of mono-substituted styrene unsaturated cardanol alkylation product is about 30%, and the average molecular weight is about 479 g/mol.

It can be seen from Fig 3 that (a) is the infrared characteristic peak of styrene unsaturated cardanol alkyl compound: O-H stretching vibration absorption peak at 3523.88 cm-1; 3019.1 cm^-1^ is the unsaturated C-H stretching vibration absorption peak; 2925.71 cm^-1^ and 2855.39 cm^-1^ are saturated C-H stretching vibration absorption peaks. The C = C stretching vibration absorption peaks at 1603.11 cm^-1^ and 1493.6 cm^-1^ in the benzene ring skeleton; 1454.03 cm^-1^ and 1411.78 cm^-1^ are the absorption peaks of alkyl C-H stretching vibration. The absorption peak of the C-O stretching vibration is at 1202.03 cm^-1^. 755.22 cm^-1^ is the benzene ring tri-substituted stretching vibration absorption peak; 820.09 cm^-1^ is the benzene ring tetra-substituted stretching vibration absorption peak. (b) Infrared characteristic peak of styrene unsaturated cardanol sulfonate compound: O-H stretching vibration absorption peak at 3505.97 cm^-1^; the absorption peak of unsaturated C-H stretching vibration is at 3027.61 cm^-1^. 2926.11 cm^-1^ and 2855.24 cm^-1^ are saturated C-H stretching vibration absorption peaks. 1606.4 cm^-1^ and 1493.22 cm^-1^ are the C = C stretching vibration absorption peaks in the benzene ring skeleton; 1454.21 cm^-1^ and 1410.14 cm^-1^ are the absorption peaks of alkyl C-H stretching vibration. 1203.73 cm^-1^ is the S = O stretching; The absorption peak of the S-O stretching vibration is at 1054.47 cm^-1^. 700.17 cm^-1^ is the benzene ring tetra-substituted stretching vibration absorption peak; 752.42 cm^-1^ is the absorption peak of benzene ring pentasubstituted stretching vibration; 629.85 cm^-1^ is the C-S stretching vibration absorption peak. The results showed that the synthesized product was MYBS.

### 2.3. Surface tension

A series of MYBS surfactant aqueous solutions with different concentrations was prepared for measurement. The surface tension experiments were carried out at different temperatures (25 °C, 45 °C, 65 °C) by the drop volume method. The γ value of water was 72.10 mN/m at 25 °C. The drop volume tube and the measuring cylinder were rinsed with distilled water and the solution to be measured three times, respectively. The extruder was controlled to inhale the solution to be measured to the scale, and the drop volume tube was lifted. One drop of the solution to be measured was extruded and suspended within 30 s to keep it from falling for 2 min. Then, the extruder was slightly adjusted to make it drop, and its volume was recorded. It was continuously measured 5 times, and the average value was taken. The CMC and γCMC values were obtained from the break point of the curve of γ and logC.

### 2.4. Conductivity measurement

The conductivity measuring instrument (model DDSJ-308F, China) is used for conductivity measurements. The titanium alloy electrode, with an electrode constant of 0.01 cm^-1^, and the sealed measuring groove are used for conductivity measurement. Before use, the conductivity measuring instrument needs to be calibrated with 0.01 mol/ L potassium chloride standard solution (conductivity is 1413 μScm^-1^) to ensure the accuracy of the measurement, and the conductivity measurement accuracy is maintained at ± 0.5%. Using a super constant temperature water bath pot (model DC0506, China), the temperature change during the experiment is controlled within ± 0.2 K. It can automatically adjust the temperature through the water cycle to maintain the constant temperature required by the conductivity meter. The conductivity (k) of aqueous solutions of MYBS surfactants at different concentrations was measured at 25 °C, 45 °C, and 65 °C. The turning point of the relationship between k and concentration (C) was regarded as the CMC [36–41].

### 2.5. Steady-state fluorescence measurements

Steady-state fluorescence spectra were recorded on an F-4600 fluorescence spectrophotometer (Hitachi High-Technologies Corp., Tokyo, Japan) at 25 ℃. Pyreneserved as the fluorescent probe at a fixed concentration of 1.0 × 10^−7^ mol/L. Emission spectra were acquired across 345–460 nm under 336 nm excitation, with slit widths set to 5.0 nm (excitation) and 2.5 nm (emission). The intensity ratio of the first (*I*_*1*_, ~ 373 nm) to third (*I*_*3*_, 384 nm) vibrational bands was analyzed to investigate surfactant aggregation behavior [42,43].

### 2.6. Dynamic light scattering test

The aggregation behavior of MYBS surfactant in aqueous solution was studied by DLS measurement on the Zetasizer Nano ZS90 dynamic light scattering instrument. The test solution was filtered through a 0.45 μm membrane filter, and the hydrodynamic radius of the MYBS surfactant at a concentration of 10 CMC was measured at a scattering angle of 90 ° and a temperature of 25 °C.

### 2.7. Transmission electron microscope

Styrene-based unsaturated cardanol sulfonate aqueous solution (10 CMC) was dropped on a carbon-coated copper TEM grid using a JEM-2100PLUS transmission electron microscope. After 5 minutes, the excess liquid was absorbed by filter paper, and then the sample was dyed with 2% (mass fraction) phosphotungstic acid solution. Remove the excess solution and dry the mesh at 25 °C.

### 2.8. Washing oil efficiency test

The 80–100 mesh quartz sand was prepared, the fine impurity components were removed by sieve screen, and then washed three times with deionized water. The dried quartz sand and crude oil were mixed and stirred evenly according to the mass ratio of 7: 1 in a 105 °C oven, and the oil sand was obtained by aging for more than 72 h in an 80 °C oven. A total of 15.0 g of aged oil sand was weighed and recorded as m. It was added to a 100 mL scale washing oil bottle, and then 30.0 mL of surfactant solution with a mass fraction of 0.3% was added. The washing oil bottle was placed in a constant temperature oscillating water bath and oscillated for 2 h under the condition of target layer temperature and oscillation frequency of 90 times/min. The washing oil bottle was taken out and supplemented with 0.3% surfactant solution at the same temperature as the washing oil bottle until the liquid level reached the top of the scale line. It was allowed to stand at the reservoir temperature for 5 h, and the volume of the washed oil was read and recorded as V (mL). The wash oil efficiency (R, %) was calculated according to Eq. (1) [44].


$$R=\frac{\rho \times \text{V}}{m\times S_{0}}\times 100\%$$ (1)

In the formula: R—Wash oil efficiency; v—volume of washout oil, mL; m—oil sand quality, g; ρ—crude oil density, g/cm^3^; S_0_—oil saturation

## 3. Results and discussion

### 3.1. Surface activity

**Fig 4:**
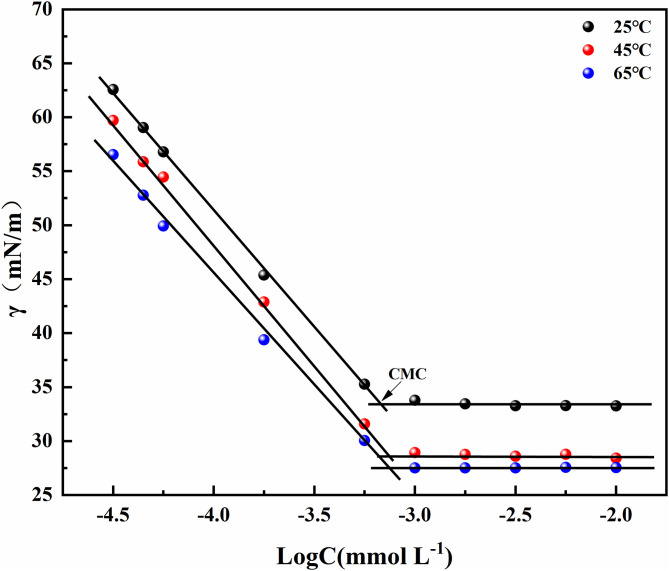
The relationship between surface tension and MYBS molar concentration at different temperatures.

The CMC and surface activity of the synthesized products were determined by the drop volume method. The curves of the surface tension of the synthesized surfactants at different temperatures with the concentration are shown in Fig 4. Other surface activity parameters are shown in Table 1. It should be noted that MYBS is treated as a mixed surfactant system obtained from synthesis rather than a conventional binary mixture of two independent surfactants; therefore, classical Clint–Rubingh analyses were not applied in the present study.

**Table 1:** Surface Property Parameters of MYBS and Other Types of Surfactants at Different Temperatures.

Surfactant	T (℃)	CMC mmol/L	γ_CMC_ mN/m	Γ_max_ μmol/m^2^	A_min_ nm^2^	pC_20_	π_cmc_ mN/m
MYBS	25	0.676	33.32	0.92	1.81	4.03	38.78
	45	0.741	28.42	0.78	2.13	4.19	43.68
	65	0.759	27.49	0.55	3.02	4.32	44.61
SDBS [45]	25	1.74^a^	36.0^a^	1.26^a^	1.32^a^	3.80^a^	36.1^a^
CDS [46]	25	5.13^b^	44.47^b^	1.08^b^	1.54^b^	2.91^b^	27.63^b^

From Fig 4, it can be seen that at 25 °C, with the increase of MYBS concentration, the γ value gradually decreases, and then tends to a constant value, where CMC is the intersection of two linear fittings: a γ steep drop area (interface adsorption dominant), a γ platform area (micelle-adsorption competitive equilibrium). As shown in Table 1, the CMC value of MYBS was 0.676 mmol/L and γ_CMC_ was 33.32 mN/m at 25 °C. The CMC and γ_CMC_ values of MYBS were lower than SDBS and CDS.

The polar heteroatoms (such as oxygen and sulfur) and rigid benzene ring groups in the molecular skeleton of surfactants play an important role in regulating intermolecular interactions, which is conducive to the formation and stability of self-assembled aggregates such as micelles [47,48]. The polar heteroatoms and rigid groups contained in exhibit multifunctional properties: for example, the spacers of surfactant molecules or the hydroxyl (-OH) groups common in the head groups are not only involved in the construction of hydrophilic head groups, but also can spontaneously embed into the interfacial transition region formed between adjacent head groups on the micelle surface [49–52]. This effect has a dual effect: first, it shields the electrostatic repulsion of adjacent positively charged groups; second, it enhances the compatibility between the hydrophobic chain and the hydrophilic head group, so that the molecules at the micelle interface are arranged closely, and the micelle stacking efficiency is improved. At the same time, the embedding of hydroxyl groups promotes the intermolecular hydrogen bond (H bond) interaction, enhances the intermolecular cohesion, accelerates the micellization process, and significantly reduces the critical micelle concentration (CMC). The rigid benzene ring group in the framework is also conducive to the formation of micelles, because the strong hydrophobicity (hydrophobic effect) and π-π interaction of the benzene ring make the molecules aggregate at a lower concentration to form micelles, and the intermolecular interaction is enhanced. Closer and more orderly arrangement is adsorbed on the gas-liquid interface [53]. Holmberg et al. [54] systematically studied the aggregation behavior of the spacers of cationic Gemini surfactants with (9E2Q-3(OH)- Q2E9,12Q-3(OH)-12Q) hydroxyl groups and without (9E2Q-3-Q2E9,12Q-3-12Q) hydroxyl groups. It was found that the CMC value of the surfactant containing a hydroxyl group was lower than that of the surfactant without a hydroxyl group. This was because the hydroxyl group shielded the electrostatic repulsion of the adjacent positively charged cations and promoted the combination of hydrogen bonds and water molecules, which made the micelles more closely arranged and improved the stability of the micelles. Similarly, the study of Yajuan Li et al. [50] showed that the CMC values of surfactants containing (φC10E5, φC10E6) benzene ring groups were lower than those of surfactants without (C_12_E_5_) benzene ring groups. This is because the rigid benzene ring group can enhance the intermolecular hydrophobic driving force and promote the formation of micelles more easily (CMC decreases). Under the π-π interaction, the aggregates are more compact and stable. At the same time, the rigid benzene ring group makes the molecular orientation more regular and the accumulation more compact at the gas-water interface, which can more effectively reduce the surface tension. Importantly, surfactants containing rigid phenyl groups improve the thermal stability and salt tolerance of the aggregates, because the π-π interaction of the rigid phenyl group can significantly enhance the intermolecular attraction, making the micelle core energy lower, the density higher, and the micelle more difficult to be destroyed at higher temperatures. At the same time, the hydrophobicity and low polarization of the molecules are enhanced, which makes the micelle core more ‘ non-polar ‘.Salt ions (such as Na ⁺ 、Cl ⁻ , and Ca²⁺) are difficult to penetrate into the micelle, and the micelle structure remains stable. This provides a significant advantage for improving oil recovery in high-temperature and high-salinity reservoirs [55].

In order to further explore the surface activity of MYBS, the maximum adsorption capacity (Γ_max_) and the minimum molecular cross-sectional area (A_min_) can be calculated. Among them, Γ_max_ can be obtained by the Gibbs adsorption isotherm equation, and A_min_ is defined as the minimum area occupied by a single surfactant molecule at the air-water interface, which together reflect the packing density of surfactant molecules at the interface. It can be deduced from the maximum surface excess adsorption capacity, Γ_max_ [56,57]:


$$\Gamma_{\text{max}}=-\frac{\text{1}}{2.303\text{nRT}}{\left(\frac{\partial_{\gamma }}{\partial_{\text{logC}}}\right)}_{\text{T}}$$ (2)

$${\text{A}}_{\text{min}}=\frac{10^{16}}{{\text{N}}_{\text{A}}\Gamma_{\text{max}}}$$ (3)

Where γ is the surface tension, the unit is mN/m, and Γ_max_ is the maximum adsorption capacity, the unit is mol/m^2^. T is the absolute temperature (298.15 K), R is the gas constant (8.314 J/mol/K), and C is the surfactant concentration, the unit is mol/L. When the surfactant concentration is lower than CMC, the slope of the linear part of the γ-logC curve is. N_A_ is the Avogadro constant (6.02 × 10^23^/mol), Amin is the minimum occupied area when the adsorption reaches saturation, and the unit is nm^2^. In this study, for anionic surfactants, n is 2 [58]. In the Table 1, the A_min_ of MYBS is greater than SDBS and CDS, because MYBS contains a rigid structure of multiple benzene rings, which increases the steric hindrance and increases the area of molecular arrangement when the interface accumulates. On the other hand, π_cmc_ is introduced to better evaluate the ability of benzene MYBS to reduce the surface tension of water, which can be calculated by Eq. (4) [59]:


$$\pi_{CMC}=\gamma_{\text{0}}-\gamma_{CMC}$$ (4)

Here, γ_0_ is the surface tension of water, and γ_CMC_ is the surface tension of MYBS solution under its CMC. The larger the π_cmc_, the higher the efficiency of the surfactant to reduce the surface tension. It can be seen that MYBS has a better ability to reduce the surface tension of water than SDBS and CDS. Further use of pC_20_ to evaluate the adsorption efficiency can be calculated using Eq. (5) [60]:


$${\text{pC}}_{20}=-{\text{logC}}_{\text{20}}$$ (5)

C_20_ is the surfactant concentration required to reduce the surface tension of water by 20 mN/m. The larger the pC_20_, the higher the efficiency of reducing the surface tension of water. The pC_20_ values of MYBS in Table 1 are higher than SDBS and CDS, and the efficiency of reducing water surface tension is the highest. This is consistent with the results obtained by π_cmc_.

### 3.2. Thermodynamic properties

**Fig 5:**
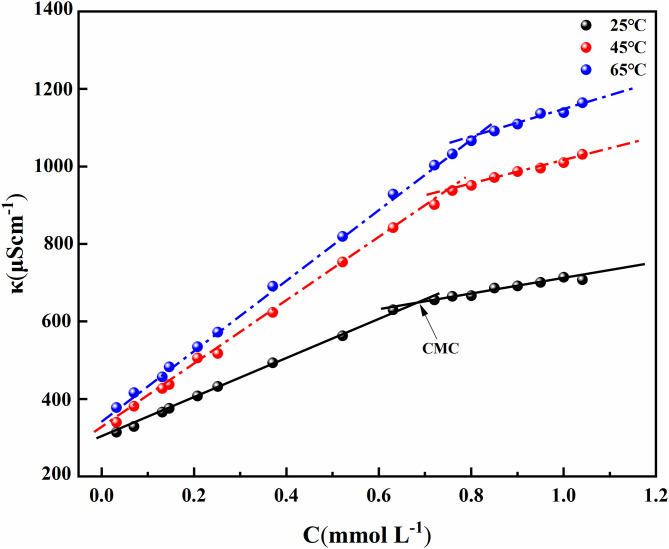
The relationship between conductivity and MYBS concentration at different temperatures.

The thermodynamic properties of MYBS surfactant were studied by measuring the electrical conductivity at different temperatures of 25 °C, 45 °C, and 65 °C. It can be seen from Fig 5 that there is a linear relationship between conductivity and concentration at each temperature. Table 2 lists the CMC values determined by the intersection of the two lines. It can be seen from Table 2 that the CMC value of MYBS surfactant increases with the increase in temperature. This is because the increase in temperature will weaken the hydration effect, resulting in the enhancement of the electrostatic repulsion of the hydrophilic group, and the micelle formation needs to overcome the larger electrostatic repulsion energy barrier, resulting in an increase in CMC. In addition, the increase in temperature intensifies the thermal motion of molecules, making it more difficult for rigid molecules to arrange to form micelles, which further leads to a significant increase in CMC.

**Table 2:** Thermodynamic Parameters of Micellization and Adsorption of MYBS at Different Temperatures.

T (℃)	CMC (mmol/L)	β	△G^o^_mic_ (KJ/mol)	△H^o^_mic_ (KJ/mol)	△S^o^_mic_ (KJ/mol/k)	T△S^o^_mic_ (KJ/mol)	△G^o^_mic_ (KJ/mol)
25	0.680	0.651	−29.85	−3.94	0.08690	25.91	−72.00
45	0.751	0.618	−30.79	−4.68	0.08207	26.11	−86.79
65	0.807	0.598	−31.99	−5.45	0.07849	26.54	−113.09

The counterion binding degree (β) can be obtained according to the following Eq. (6) [61]:


$$\beta =1-\frac{S_{micellar}}{S_{premicellar}}$$ (6)

S_micellar_ and S_premicellar_ are the slopes of the linear part of k and C before and after CMC, respectively. The value of β is listed in Table 2, which decreases with the increase in temperature. This is due to the decrease in the electrostatic attraction of the micelle surface to the counterions, indicating that the polymerization of the counterions and the micelle surface is an exothermic process [62]. The thermodynamic parameters of micellization can be calculated by the following equation [63,64], and the corresponding results are listed in Table 2.


$$\Delta {{\text{G}}^{\text{o}}}_{\text{mic}}=\text{RT}(1+\beta )\text{lnCMC}$$ (7)

$$\Delta {{\text{H}}^{\text{o}}}_{\text{mic}}=-{\text{RT}}^{\text{2}}(1+\beta )\frac{\alpha_{ln\text{CMC}}}{\alpha_{T}}$$ (8)

$$\Delta {{\text{S}}^{\text{o}}}_{\text{mic}}=\frac{\Delta {{\text{H}}^{\text{o}}}_{\text{mic}}-\Delta {{\text{G}}^{\text{o}}}_{\text{mic}}}{\text{T}}$$ (9)

$$\Delta {{\text{G}}^{\text{o}}}_{\text{ads}}=\Delta {{\text{G}}^{\text{o}}}_{\text{mic}}-\frac{\pi_{\text{CMC}}}{\Gamma_{\text{max}}}$$ (10)

Where $\Delta {{\text{H}}^{\text{o}}}_{\text{mic}}$ is the standard enthalpy change of micelle formation, $\Delta {{\text{S}}^{\text{o}}}_{\text{mic}}$ is the standard entropy change of micellization process, $\Delta {{\text{G}}^{\text{o}}}_{\text{mic}}$ and $\Delta {{\text{G}}^{\text{o}}}_{\text{ads}}$ are the standard Gibbs free energy changes of micellization and adsorption, respectively. T is the absolute temperature and R is the gas constant.

In Table 2, all $\Delta {{\text{G}}^{\text{o}}}_{\text{mic}}$ and $\Delta {{\text{G}}^{\text{o}}}_{\text{ads}}$ values are negative, indicating that the micellization and adsorption processes of synthetic surfactants are spontaneous [65]. In this case, the negative value of $\Delta {{\text{G}}^{\text{o}}}_{\text{ads}}$ is more negative than the corresponding value of $\Delta {{\text{G}}^{\text{o}}}_{\text{mic}}$, indicating that adsorption is the main process relative to micellization [66]. With the increase in temperature, the absolute values of $\Delta {{\text{G}}^{\text{o}}}_{\text{mic}}$ and $\Delta {{\text{G}}^{\text{o}}}_{\text{ads}}$ gradually increase. Therefore, the increase of temperature is beneficial to the micellization and adsorption of molecules. At the same time, the absolute value of $\Delta {{\text{G}}^{\text{o}}}_{\text{ads}}$ is much larger than the absolute value of $\Delta {{\text{G}}^{\text{o}}}_{\text{mic}}$, indicating that the surface active molecules prefer to adsorb at the interface rather than form micelles [67].

The $\Delta {{\text{H}}^{\text{o}}}_{\text{mic}}$ values of MYBS surfactants are all negative, indicating that the micellization and adsorption are exothermic processes. All $\Delta {{\text{S}}^{\text{o}}}_{\text{mic}}$ values are positive, which means that the micellization process will increase the degree of molecular disorder. It is possible that when a small amount of synthetic surfactants is dissolved in water, the hydrogen bond structure between water molecules will be rearranged and a new structure (i.e., iceberg structure) different from the water structure will be formed around the hydrophobic carbon chain [68]. When the concentration of the synthetic surfactant reaches CMC, the hydrophobic carbon chains will approach and aggregate with each other, thus destroying the iceberg structure and increasing the disorder degree of the surfactant solution [69,70]. On the other hand, it can be seen from Table 2 that $\Delta {{\text{S}}^{\text{o}}}_{\text{mic}}$ is significantly larger than $\Delta {{\text{H}}^{\text{o}}}_{\text{mic}}$, which means that the main contribution of $\Delta {{\text{G}}^{\text{o}}}_{\text{mic}}$ comes from $\Delta {{\text{S}}^{\text{o}}}_{\text{mic}}$ in the process of micelle formation. Therefore, the micellization of MYBS surfactant is an entropy-driven process in the studied temperature range.

### 3.3. Micropolarity

**Fig 6:**
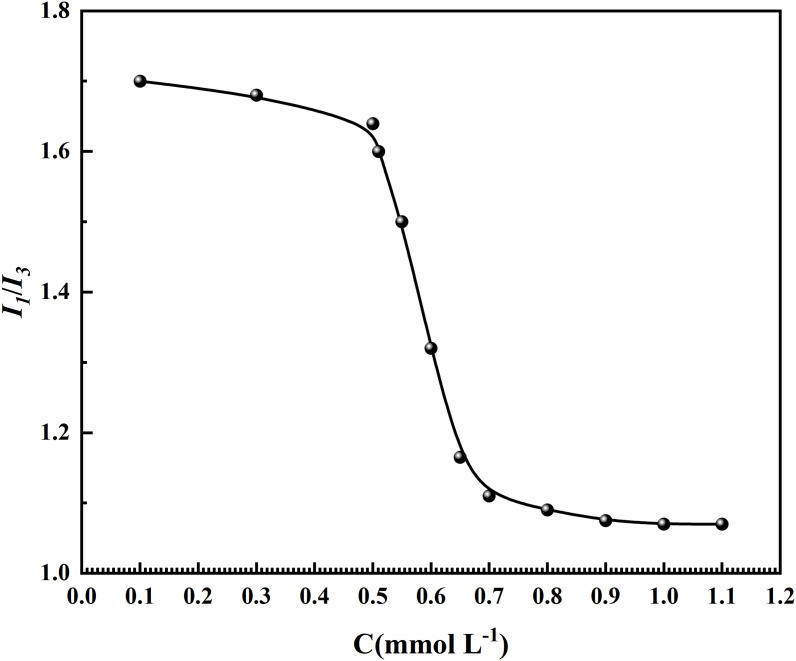
Variation of the Pyrene intensity ratio (*I*_*1*_/*I*_*3*_)vs. the concentrations (C)for the MYBS at 25 ℃.

Pyrene serves as a widely employed hydrophobic probe in aqueous systems due to its negligible water solubility (effectively insoluble, withan *I*_*1*_/*I*_*3*_ ratio of ~ 1.8 in pure water) [71]. The CMC values of surfactants are identified by monitoring the abrupt transition in the *I*_*1*_/*I*_*3*_ ratio as pyrene enters the micellar structures [72]. Fig 6 demonstrates the characteristic sigmoidal variation of *I*_*1*_/*I*_*3*_ ratios vs. concentration for MYBS surfactants. The curve of the change in the values of *I*_*1*_/*I*_*3*_ with the concentration of MYBS are shown in Fig 6. The CMC value of MYBS derived from Boltzmann fitting [71,73] is 0.672 mmol/L, consistent with findings from both surface tension and conductivitymethods.

### 3.4. Aggregation behavior

The aggregate structure of anionic surfactant aggregates can be described by the Tanford equation [74], which can be used for anionic surfactants [75,76].


$$P=\frac{V}{a_{0}\times l_{0}}$$ (11)

$$V=(27.4+26.9m)\times 10^{-3}{nm}^{3}$$ (12)

$$l_{0}=(0.15+0.1265m)nm$$ (13)

Where *P* is the stacking parameter, *l*_*0*_ and *V* are the length and hydrophobic volume of the alkyl chain, respectively. The calculated *l*_*0*_ and *V* should be regarded as approximate parameters used for trend analysis rather than strictly quantitative structural descriptors. *m* is thenumber of carbon atoms in the hydrophobic alkyl chain, calculated by the formula *m* = 0.7 *m*_*1*_ + 0.3 *m*_*2*_, and *a*_*0*_ is the minimum average occupied area of surfactant molecules. According to the packing parameter theory, when *P* ≤ 0.5, the surfactant molecules tend to form small spherical micelles [74,77].

**Fig 7:**
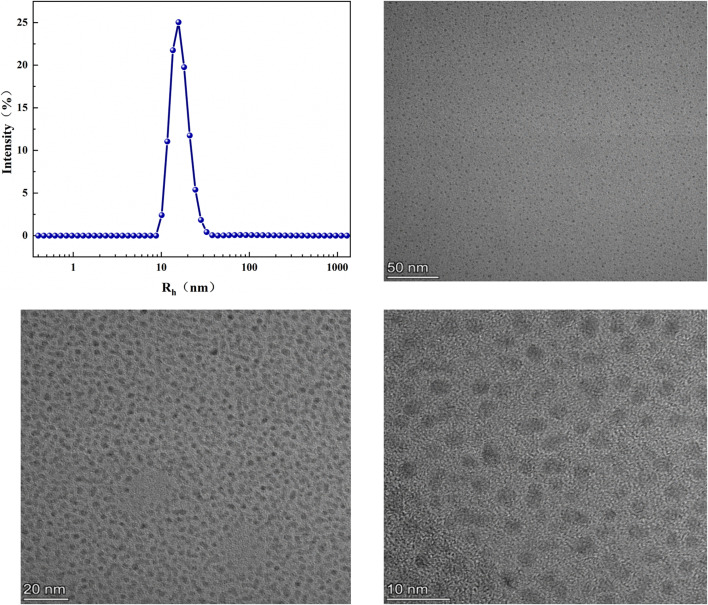
Size distributions from DLS measurements and TEM micrographs size distributions of the aqueous solution of MYBS (10 CMC).

The *P* value of MYBS surfactant in Table 3 indicates that MYBS can aggregate to form small spherical micelles. The aggregation morphology and particle size distribution of these aggregates were further confirmed by DLS and TEM. As shown in Fig 7 DLS measurement and TEM aggregation morphology, MYBS surfactant has an obvious peak in the range of 10 nm-30 nm, indicating the existence of small spherical micelles in aqueous solution. Because the MYBS surfactant contains long hydrophobic alkyl chains and polycyclic aromatic hydrocarbon groups, it has strong micelle formation ability, and intramolecular association occurs, which makes the spherical micelle curvature formed by the aggregation of the surfactant larger and the micelle size smaller. This is consistent with the P value calculated by the Tanford expression. In order to intuitively understand the above results, the aggregation morphology of MYBS surfactant at 10 CMC was observed by transmission electron microscopy (TEM). The small spherical micelle structure of MYBS can be clearly observed. This is consistent with the prediction results of the accumulation parameter theory. The larger micellar size obtained from DLS compared with TEM can be attributed to the presence of the hydration shell and the dynamic nature of micelles in solution, which is a common observation in surfactant systems. The difference between DLS and TEM sizes arises from the distinct physical principles of the two techniques, and the consistency in morphology and size scale confirms the formation of spherical micelles.

**Table 3:** Packing Parameter *P* and Other Physicochemical Parameters(*V* and *l*_*0*_).

Surfactant	*V*	*l*_o_(nm)	*P*
MYBS	0.931	4.526	0.25

The DLS and TEM results consistently confirm the formation of small spherical micelles, which is in agreement with the predicted aggregation behavior based on the packing parameter. These findings, combined with thermodynamic and interfacial analyses, provide a comprehensive understanding of the micellization process of MYBS. Related interfacial and aggregation behaviors have also been discussed in a broader interdisciplinary context, including biological and immunological interfaces [78,79]. This broader perspective highlights the relevance of our findings beyond conventional surfactant systems and may inform future applications in complex microenvironments.

### 3.5. Washing oil efficiency

The effect of surfactant solution with a mass fraction of 0.3% on the efficiency of crude oil washing was studied. The test results are shown in Table 4.

**Table 4:** Washing Oil Efficiency Test Results.

Surfactant	Mass fraction/%	Oil washing efficiency/%
MYBS	0.3	73
SDBS	0.3	30

It can be seen from Table 4 that the oil washing efficiency of MYBS surfactant with a mass fraction of 0.3% is 73%, which is much higher than that of SDBS surfactant with a mass fraction of 0.3%. This is because the MYBS surfactant contains a multi-benzene ring and a longer hydrophobic alkyl chain structure, which can be fully embedded in the crude oil droplets, reducing the adhesion of crude oil and sand, making it easier to peel off the adsorbed crude oil on the surface of the oil sands, thereby significantly improving the oil washing efficiency.

## 4. Conclusion

A mixed cashew phenol sulfonate surfactant (MYBS) of disubstituted styrene unsaturated cashew phenol sulfonate (2,4-YBS) and monosubstituted styrene unsaturated cashew phenol sulfonate (2-YBS) was synthesized. The molar percentage content of the alkylated product was determined to be 70%: 30% by gas chromatography-mass spectrometry, and the average molecular weight was 479 g/mol. The structure of the molecule was characterized by infrared spectroscopy. The surface activity of MYBS surfactant is higher than SDBS and CDS [45,46]. Thermodynamic parameters indicate that the micellization process is spontaneous and entropy-driven. Transmission electron microscopy and dynamic light scattering measurements confirmed that the MYBS surfactant formed small spherical micelles with micelle sizes ranging from 10 nm to 30 nm, which was consistent with the predicted results based on the stacking parameter P value. Therefore, the research results are helpful to expand the understanding of the physical and chemical properties and structure-performance relationship of the current cardanol anionic surfactants. At the same time, the oil washing efficiency test result is 73%, which has a good oil washing effect. Compared with previously reported cardanol-based and aromatic surfactants [17,19]. The MYBS surfactants developed in this work exhibit enhanced interfacial efficiency and well-defined aggregation behavior, supported by complementary interfacial, spectroscopic, and structural characterizations. This study combines basic science with practical energy applications to advance the field towards more efficient and environmentally friendly solutions for enhanced oil recovery.

The present study focuses on MYBS as a mixed surfactant system, and direct quantitative comparisons with individual components were beyond the scope of this work. In the future work, Quantitative evaluation of ideality in well-defined binary mixtures will be considered. A systematic comparison between MYBS and its individual components (e.g., 2-YBS and 2,4-YBS) to further elucidate structure–property relationships.

## Supporting information

S1 File Original data for surface tension, conductivity, fluorescence, and oil washing efficiency experiments. (DOC)
