# Supplementary material for: Synthesis, surface activities, aggregation properties and oil washing performances of novel cardanol-based surfactants
Source: PLoS One. 2026 Apr 22;21(4):e0344255. doi: 10.1371/journal.pone.0344255 (PMC13102204; doi:10.1371/journal.pone.0344255)
Supplement: S1 File — (DOC) [file pone.0344255.s001.doc]

**Supporting Information**

**Original data for surface tension, conductivity, fluorescence, and oil washing efficiency experiments.**

**S1 Table. Raw surface tension measurements of MYBS solutions at different temperatures, corresponding to Fig 4 Each data point represents an independent measurement (n=3).**

**(25℃)**

| **Concentration (mmol·L⁻¹)** | **γ₁**  **(mN·m⁻¹)** | **γ₂** | **γ₃** | **Mean ± SD** |
| --- | --- | --- | --- | --- |
| **10.0** | **33.28** | **33.26** | **33.27** | **33.27±0.01** |
| **5.62** | **33.29** | **33.31** | **33.28** | **33.29±0.01** |
| **3.16** | **33.31** | **33.26** | **33.25** | **33.27±0.03** |
| **1.78** | **33.42** | **33.55** | **33.37** | **33.45±0.02** |
| **1.00** | **33.85** | **33.82** | **33.68** | **33.78±0.06** |
| **0.562** | **35.35** | **35.35** | **35.12** | **35.27±0.06** |
| **0.178** | **45.38** | **45.39** | **45.37** | **45.38±0.01** |
| **0.0562** | **56.79** | **56.54** | **57.05** | **56.78±0.36** |
| **0.0447** | **59.05** | **58.85** | **59.22** | **59.04±0.30** |
| **0.0316** | **62.57** | **62.58** | **62.56** | **62.57±0.01** |

**(45℃)**

| **Concentration (mmol·L⁻¹)** | **γ₁**  **(mN·m⁻¹)** | **γ₂** | **γ₃** | **Mean ± SD** |
| --- | --- | --- | --- | --- |
| **10.0** | **28.43** | **28.42** | **28.44** | **28.43±0.01** |
| **5.62** | **28.78** | **28.77** | **28.76** | **28.78±0.01** |
| **3.16** | **28.59** | **28.60** | **28.58** | **28.59±0.01** |
| **1.78** | **28.77** | **29.10** | **28.45** | **28.77±0.52** |
| **1.00** | **28.95** | **28.97** | **28.88** | **28.93±0.07** |
| **0.562** | **31.59** | **31.58** | **31.60** | **31.59±0.01** |
| **0.178** | **42.89** | **42.93** | **42.85** | **42.89±0.06** |
| **0.0562** | **54.43** | **54.46** | **54.50** | **54.46±0.04** |
| **0.0447** | **55.85** | **55.90** | **55.86** | **55.87±0.05** |
| **0.0316** | **59.70** | **59.71** | **59.69** | **59.70±0.01** |

**(65℃)**

| **Concentration (mmol·L⁻¹)** | **γ₁**  **(mN·m⁻¹)** | **γ₂** | **γ₃** | **Mean ± SD** |
| --- | --- | --- | --- | --- |
| **10.0** | **27.49** | **27.55** | **27.61** | **27.55±0.06** |
| **5.62** | **27.57** | **27.58** | **27.56** | **27.57±0.01** |
| **3.16** | **27.53** | **27.52** | **27.51** | **27.52±0.01** |
| **1.78** | **27.52** | **27.53** | **27.51** | **27.52±0.01** |
| **1.00** | **27.61** | **27.50** | **27.45** | **27.52±0.09** |
| **0.562** | **30.28** | **29.87** | **29.91** | **30.05±0.29** |
| **0.178** | **39.38** | **39.39** | **39.37** | **39.38±0.01** |
| **0.0562** | **49.95** | **49.93** | **49.94** | **49.94±0.01** |
| **0.0447** | **52.80** | **52.78** | **52.76** | **52.78±0.02** |
| **0.0316** | **56.53** | **56.55** | **56.54** | **56.54±0.01** |

**S2 Table. Raw conductivity measurements of MYBS aqueous solutions at different temperatures, corresponding to Fig 5.**

| **Concentration (mmol·L⁻¹)** | **κ (μS·cm⁻¹) @25 ℃** | **κ**  **@45 ℃** | **κ**  **@65 ℃** |
| --- | --- | --- | --- |
| **1.04** | **707.78** | **1031.48** | **1164.62** |
| **1.00** | **714.24** | **1010.08** | **1139.15** |
| **0.95** | **701.00** | **995.81** | **1137.11** |
| **0.90** | **691.82** | **986.98** | **1109.60** |
| **0.85** | **686.39** | **971.70** | **1091.59** |
| **0.80** | **666.69** | **951.32** | **1066.46** |
| **0.76** | **664.93** | **938.07** | **1032.49** |
| **0.72** | **655.82** | **901.73** | **1003.62** |
| **0.63** | **630.34** | **842.29** | **928.90** |
| **0.52** | **563.09** | **753.64** | **819.53** |
| **0.37** | **493.35** | **623.55** | **691.08** |
| **0.25** | **432.32** | **517.58** | **572.60** |
| **0.21** | **407.87** | **506.03** | **534.90** |
| **0.15** | **375.98** | **437.42** | **483.05** |
| **0.13** | **365.95** | **427.23** | **457.12** |
| **0.07** | **329.07** | **381.38** | **416.47** |
| **0.03** | **314.46** | **339.94** | **378.04** |

**S3 Table. Ratio of original fluorescence intensity of pyrene in MYBS solution at 25℃ ( *I_1_ / I_3_* ), corresponding to Fig 6.**

| **Concentration**  **(mmol·L⁻¹)** | ***I_1_ / I_3_*** |
| --- | --- |
| **0.1** | **1.70** |
| **0.3** | **1.68** |
| **0.5** | **1.64** |
| **0.51** | **1.60** |
| **0.55** | **1.50** |
| **0.60** | **1.32** |
| **0.65** | **1.17** |
| **0.70** | **1.11** |
| **0.80** | **1.09** |
| **0.90** | **1.08** |
| **1.00** | **1.07** |
| **1.10** | **1.07** |

**S4 Table. Raw data for oil washing efficiency experiments using MYBS and SDBS surfactant solutions, corresponding to Table 4. Each experiment was conducted in triplicate.**

1. **Raw data for oil washing efficiency experiments using MYBS surfactant solutions**

| **Run** | **Oil type** | **Initial oil mass**  **(g)** | **Residual oil mass**  **(g)** | **Removal efficiency(%)** |
| --- | --- | --- | --- | --- |
| **1** | **oil samples from Liaohe Oilfield, China** | **1.875** | **1.369** | **73.00** |
| **2** | **oil samples from Liaohe Oilfield, China** | **1.875** | **1.370** | **73.06** |
| **3** | **oil samples from Liaohe Oilfield, China** | **1.875** | **1.368** | **72.96** |

1. **Raw data for oil washing efficiency experiments using SDBS surfactant solutions**

| **Run** | **Oil type** | **Initial oil mass**  **(g)** | **Residual oil mass**  **(g)** | **Removal efficiency(%)** |
| --- | --- | --- | --- | --- |
| **1** | **oil samples from Liaohe Oilfield, China** | **1.875** | **0.563** | **30.00** |
| **2** | **oil samples from Liaohe Oilfield, China** | **1.875** | **1.370** | **30.31** |
| **3** | **oil samples from Liaohe Oilfield, China** | **1.875** | **1.368** | **29.71** |
